# Prevalence of and Risk Factors for Post–COVID-19 Condition during Omicron BA.5–Dominant Wave, Japan

**DOI:** 10.3201/eid3007.231723

**Published:** 2024-07

**Authors:** Arisa Iba, Mariko Hosozawa, Miyuki Hori, Yoko Muto, Isao Muraki, Rie Masuda, Nanako Tamiya, Hiroyasu Iso

**Affiliations:** National Center for Global Health and Medicine, Tokyo, Japan (A. Iba, M. Hosozawa, M. Hori, Y. Muto, H. Iso);; Osaka University, Osaka, Japan (I. Muraki);; University of Tsukuba, Ibaraki, Japan (R. Masuda, N. Tamiya)

**Keywords:** COVID-19, post–acute COVID-19 syndrome, post–COVID-19 condition, prevalence, risk factors, SARS-CoV-2, severe acute respiratory syndrome coronavirus 2, viruses, respiratory infections, zoonoses, Japan, Omicron, BA.5

## Abstract

The increased risk for post–COVID-19 condition after the Omicron-dominant wave remains unclear. This population-based study included 25,911 persons in Japan 20–69 years of age with confirmed SARS-CoV-2 infection enrolled in the established registry system during July–August 2022 and 25,911 age- and sex-matched noninfected controls who used a self-reported questionnaire in January–February 2023. We compared prevalence and age- and sex-adjusted odds ratios of persistent COVID-19 symptoms (lasting ≥2 months). We evaluated factors associated with post–COVID-19 condition by comparing cases with and without post–COVID-19 condition. We analyzed 14,710 (8,392 cases and 6,318 controls) of 18,183 respondents. Post–COVID-19 condition proportion among cases was 11.8%, higher by 6.3% than 5.5% persistent symptoms among controls. Female sex, underlying medical conditions, mild to moderate acute COVID-19, and vaccination were associated with post–COVID-19 condition. Approximately 12% had post–COVID-19 condition during the Omicron-dominant wave, indicating the need for longer follow-up.

COVID-19 has caused a significant global disease burden since it was first identified in December 2019; as of May 2024, >750 million cases had been confirmed, and ≈7.5 million deaths had occurred worldwide (*1*). In addition to acute illnesses, the prolonged or recurrent symptoms occurring after an initial infection SARS-CoV-2, referred to as post–COVID-19 condition (*2*), have also raised concerns.

More than 65 million persons worldwide have post–COVID-19 condition (*3*). On the basis of estimates of those infected during March 2020–November 2021, a total of 10%–30% of nonhospitalized case-patients and 50%–70% of hospitalized case-patients have had post–COVID-19 condition. Frequently reported symptoms included fatigue, dyspnea, neurocognitive impairment, and loss of smell in patients infected during January 2020–August 2021 (*4**–**8*). The risk of developing post–COVID-19 condition was higher in female patients, those with severe acute COVID-19, or those with a greater number of acute symptoms (*4*,*7*,*9*,*10*). We noted those results in patients infected with variants before the Omicron variant emerged.

The Omicron variant was identified in November 2021; the BA.5 lineage of that variant was detected in April 2022 and has since spread worldwide. The Omicron variant tends to cause less severe acute symptoms (*11*) and has a similar or lower risk for post–COVID-19 condition than the previous variants (*12*–*16*). However, most previous studies concerning post–COVID-19 condition in relation to the Omicron variant, except those that used electronic health record data (*17*), were hospital-based (*13*–*15*,*18*–*21*) or population-based without a control group (*12*,*16*,*22*,*23*). Longer sequelae and risks for post–COVID-19 condition in persons infected with the Omicron variant compared with noninfected populations remain unknown. As the number of COVID-19 cases has increased, with greater infectivity of the Omicron variant (*24*) in addition reductions in nonpharmaceutical interventions (e.g., lockdowns, social distancing, mask requirements), it is crucial to investigate the potential long-term consequences of infection with the Omicron variant. We conducted a population-based study of symptoms after acute COVID-19 using a self-reported questionnaire in a large city in Japan. Our objective was to examine the increased risk for persistent symptoms after SARS-CoV-2 infection compared with a noninfected population, focusing specifically on the Omicron variant (especially the BA.5 lineage). We also investigated the factors associated with post–COVID-19 condition.

## Methods

### Study Design and Participants

We conducted a population-based study of community-dwelling adults 20–69 years of age who had confirmed SARS-CoV-2 infection during July–August 2022. We extracted data from the Japan Health Center Real-time Information-sharing System on COVID-19 (HER-SYS), the established registry system, and age- and sex-matched controls using a self-reported web-based questionnaire in Shinagawa City, a metropolitan area located in the Tokyo area of Japan. The population of Shinagawa City is ≈400,000 and its population density is 17,700 persons/km^2^.

Japan experienced the 7th wave of COVID-19 in July 2022, caused by the Omicron subvariant BA.5 lineage. The prevalence of the BA.5 lineage increased from 67% in epidemiologic week 27 (July 7–10, 2022) to 92% in epidemiologic week 30 (July 25–31, 2022), becoming dominant (*25*). When COVID-19 was diagnosed by a positive reverse transcription PCR or a lateral flow antigen test for SARS-CoV-2 or a clinical diagnosis (for symptomatic close contacts), the attending physician was required to document every case in HER-SYS until September 26, 2022. Patients needed to see a physician to undergo a test for SARS-CoV-2 until the Ministry of Health, Labour, and Welfare approved over-the-counter antigen test kits on August 24, 2022. However, most patients visited a physician even after the over-the-counter antigen test kits became available rather than testing themselves at home. Therefore, most of the infected persons were registered in HER-SYS during the study period.

**Figure 1:**
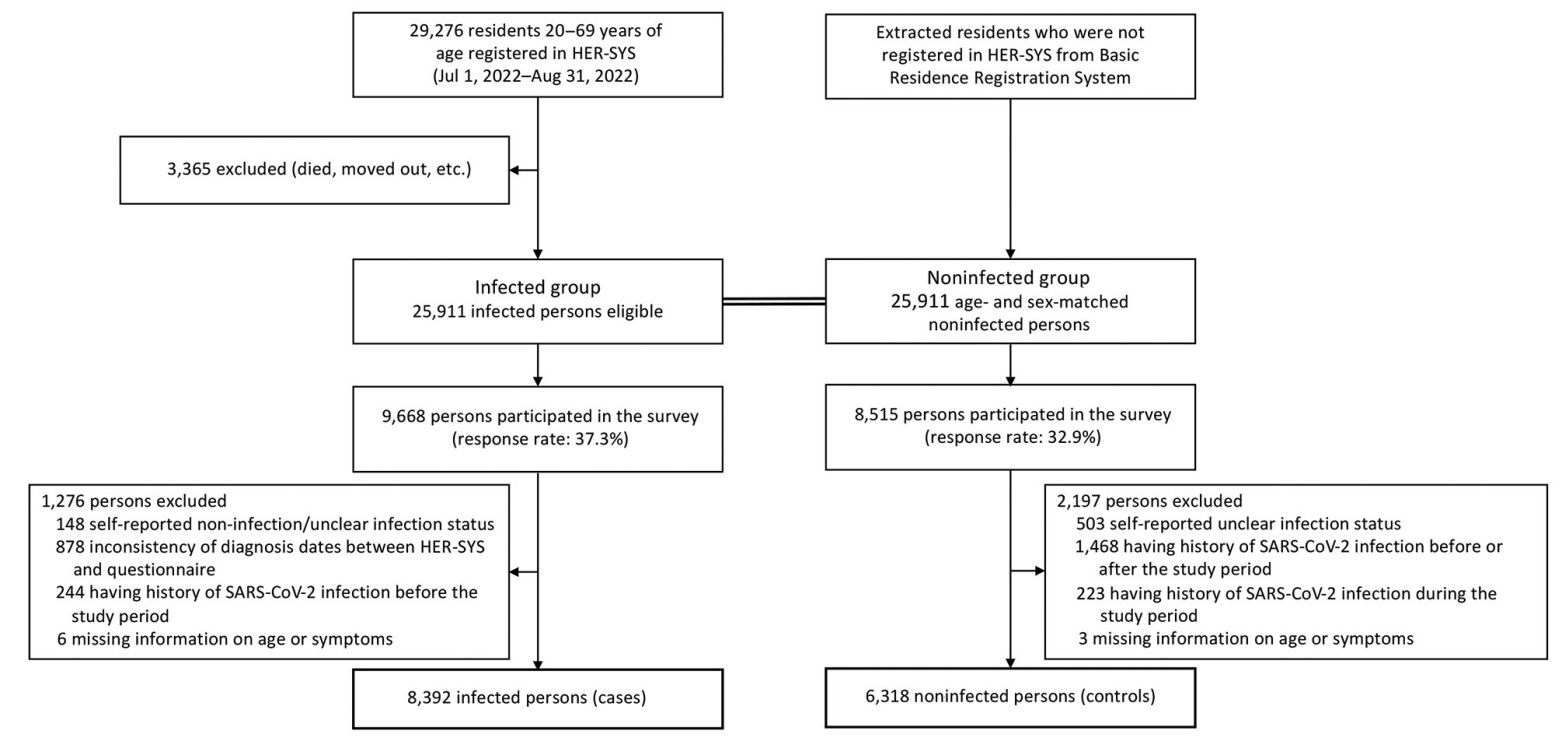
Flowchart of participant selection in study of prevalence and risk factors for post–COVID-19 condition during Omicron BA.5–dominant wave, Japan. Of 29,276 residents 20–69 years of age identified in the municipal HER-SYS database as infected with COVID-19, we selected a total of 25,911 participants; we extracted the same number of age- and sex-matched noninfected residents from the Basic Residence Registration System to serve as the control group. HER-SYS, Health Center Real-time Information-sharing System on COVID-19.

We selected participants registered in the HER-SYS database who were 20–69 years of age and infected with SARS-CoV-2 during July 1–August 31, 2022. We excluded 3,365 of the 29,276 identified infected residents who had died or moved out of the area and selected the remaining 25,911 infected persons as study participants (infected group). We matched data from HER-SYS and the Basic Resident Registration system (the municipal residence record of the name, birthdate, sex, and address of all residents living in a municipality) to identify noninfected residents who had never been registered in the HER-SYS database during the participant selection. We selected 25,911 age- and sex-matched noninfected persons (noninfected group) from the matched dataset (Figure 1). The ethics committee of the National Center for Global Health and Medicine approved this study (NCGM-S-004571).

We sent research information and invitations to the online questionnaire to the selected participants (25,911 each in the infected and noninfected group) by mail on January 11–13, 2023, approximately 6 months after infection for those who had COVID-19 (cases). Respondents were required to provide consent to participate in the study before accessing the website; those who agreed answered the questionnaire by February 13. At the beginning of the questionnaire, we asked participants if they had a diagnosis of COVID-19. If they answered “yes,” they were directed to the questions for infected persons, which inquired about the number and date of infection episodes. If they answered “no,” “I don’t know,” or “I prefer not to answer,” they were directed to the questions for noninfected persons (Appendix). We included persons whose answers on infection status were consistent with HER-SYS data and whose first infection was within the study period.

### Post–COVID-19 Condition (Cases) and Persistent Symptoms (Controls)

We asked the participants about the presence of 26 symptoms that emerged during or after the first SARS-CoV-2 infection for cases and in July 2022 for controls. The symptoms were selected from the International Severe Acute Respiratory and Emerging Infection Consortium COVID-19 questionnaire. Symptoms were fever, cough, fatigue, sore throat, chest pain, anorexia, brain fog, difficulty concentrating, anosmia, ageusia, shortness of breath, hair loss, muscle weakness, palpitations, sleep disorder, rhinorrhea, headache, joint pain and swelling, muscle aches, nausea/vomiting, abdominal pain, skin rash, eye-related symptoms, dizziness, erectile dysfunction (male only), and menstrual change (female only) (*26*). If a symptom was present, we asked about its timing and duration: whether they had the symptom at illness onset or 3 months after infection (infected group only), whether they had it at the time of the survey, and whether the symptom persisted for ≥2 months. For those who affirmed they had any symptoms, we asked the extent to which the symptoms hindered daily life at the time of response using an 11-point scale from 0 (no effect) to 10 (extreme hindrance) and categorized those responses into 4 levels: 0, no effect; 1–3, mild hindrance; 4–6, moderate hindrance; and 7–10, serious hindrance.

For cases, we defined post–COVID-19 condition based on the World Health Organization (WHO) definition (*27*): a symptom that persisted for >2 months after the acute phase. For brain fog, difficulty concentrating, hair loss, and muscle weakness, we defined post–COVID-19 condition as symptoms having lasted >2 months during the observation period regardless of the timing because those symptoms develop in the subacute phase (*17*,*28*). For controls, we defined persistent symptoms as symptoms lasting >2 months experienced between July 2022 and the date of the survey.

### Variables

We asked infected persons about the severity of acute COVID-19 and categorized them into 4 groups according to the WHO clinical severity scale: asymptomatic, mild (symptomatic but not admitted to the hospital), moderate (admitted to the hospital, required supplemental oxygen, or both), and severe (received mechanical ventilation or intensive care admission) (*29*). We counted the number of infections because some participants had been infected >1 time during the observation period. We also asked participants about their demographics (i.e., age at the answering date, sex, height, and weight), underlying medical conditions before the infection (or before July 2022 in the noninfected group), lifestyle, and socioeconomic status (e.g., household income and educational level). We calculated equivalized household income by dividing household income by the square root of the household size. For vaccination status, we extracted the vaccination date, vaccination type, and number of vaccinations from the municipality’s Vaccination Record System. We substituted the questionnaire responses for missing values for 1,589 (10%) respondents (e.g., those who had moved from the original municipality).

### Statistical Analysis

We determined the participants’ characteristics according to their infection status and compared using the *t*-test for continuous variables and χ^2^ test for categorical variables. We calculated the proportions of overall and each post–COVID-19 condition (cases) and persistent symptoms (controls). Using multivariable logistic regression analysis, we calculated the age- and sex-adjusted odds ratios of each symptom in the cases compared with the persistent symptoms in the controls as a reference. We also investigated the risk factors associated with post–COVID-19 condition among cases using multivariate logistic regression models. Model 1 comprised age group and sex; model 2, underlying medical conditions, body mass index, severity, and vaccination status before infection; and model 3, household income and educational level. We conducted multiple imputations using chained equations to account for missing data in model 3; the proportion of missing values in household income was 13.1%. We included all explanatory and outcome variables in the imputation model to create 50 imputed datasets. We also calculated the proportion of influence of post–COVID-19 condition on daily life. We defined statistical significance as a 2-sided p value <0.05. We used Stata version 17 MP software (StataCorp LLC, https://www.stata.com) for all analyses.

## Results

A total of 51,822 persons were invited to participate in the study, of whom 18,183 responded to the questionnaire (response rate 35.1%). The response rate was higher in the infected group than in the noninfected group (37.3% vs. 32.9%, difference of 4.4% [95% CI 3.0%–5.8%]). The response rate was higher among female than male persons in all age groups of both infected and noninfected groups. Among male invitees, the difference in response rates between the infected and noninfected groups was large for age groups in their 50s (12.8% [95% CI 8.1%–17.5%]) and 60s (8.5% [95% CI 1.6%–15.4%]) (Table 1).

**Table 1:** Response rates of persons in study of prevalence and risk factors for post–COVID-19 condition during BA.5 Omicron-dominant wave, Japan*

Age group, y	Infected persons					Noninfected persons					Difference in response rates (95% CI)
	HER-SYS†	No. participants	No. responses	Response rate, %		BRRS‡	No. participants	No. responses	Response rate, %		
Male patients											
20–29	3,404	2,979	611	20.5		3,404	2,979	574	19.3		1.2 (–3.3 to 5.7)
30–39	3,806	3,328	1,120	33.7		3,806	3,328	896	26.9		6.8 (2.8–10.8)
40–49	3,461	3,058	1,061	34.7		3,461	3,058	943	30.8		3.9 (–0.2 to 8.0)
50–59	2,586	2,243	923	41.2		2,586	2,243	636	28.4		12.8 (8.1–17.5)
60–69	1,152	1,024	413	40.3		1,152	1,024	326	31.8		8.5 (1.6–15.4)
Subtotal	14,409	12,632	4,129	32.7		14,409	12,632	3,375	26.7		6.0 (3.9–8.1)
Female patients											
20–29	3,682	3,218	960	29.8		3,682	3,218	912	28.3		1.5 (–2.6 to 5.6)
30–39	4,028	3,582	1,565	43.7		4,028	3,582	1,491	41.6		2.1 (–1.4 to 5.6)
40–49	3,671	3,313	1,554	46.9		3,671	3,313	1,423	43.0		3.9 (3.2–7.5)
50–59	2,386	2,166	971	44.8		2,386	2,166	884	40.8		4.0 (–0.5 to 8.5)
60–69	1,100	1,000	402	40.2		1,100	1,000	333	33.3		6.9 (–0.1 to 13.9)
Subtotal	14,867	13,279	5,456	41.1		14,867	13,279	5,047	38.0		3.1 (1.2–5.0)
Total	29,276	25,911	9,668	37.3		29,276	25,911	8,515	32.9		4.4 (3.0–5.8)

*BRRS, Basic Resident Registration system; HER-SYS, Health Center Real-Time Information-Sharing System. †Numbers of SARS-CoV-2–infected persons extracted from the HER-SYS database. ‡Numbers of age- and sex-adjusted noninfected persons extracted from the Basic Resident Registration system database.

We excluded 3,473/18,183 respondents for responses of infectious status inconsistent with HER-SYS (answering different infection statuses or different diagnosis date) and reporting a prior infection and 9 because their records were missing data on age or symptoms. A total of 14,710 participants (8,392 cases and 6,318 controls) were eligible for the analysis (Figure 1). Mean age of all participants was 42.4 (SD 11.7) years; 8,502 (57.8%) participants were female and 6,087 (41.4%) male (Table 2). Mean age of case participants was 42.3 (SD 11.6) years; 4,802 (57.2%) case participants were female and 3,535 (42.1%) male. The mean follow-up period from SARS-CoV-2 infection to the response date was 167.9 (SD 14.5) days. Most cases (8,326 [99.2%] patients) demonstrated asymptomatic to mild disease, whereas 66 (0.8%) cases had moderate to severe disease.

**Table 2:** Characteristics of participants in study of prevalence and risk factors for post–COVID-19 condition during BA.5 Omicron-dominant wave, Japan*

Characteristic	Cases, n = 8,392		Controls, n = 6,318		p value
Mean age, y (+SD)	42.3 (+11.6)		42.4 (+11.8)		0.63
Age group, y					0.29
20–29	1,316 (15.7)		1,036 (16.4)		
30–39	2,340 (27.9)		1,674 (26.5)		
40–49	2,326 (27.7)		1,766 (28.0)		
50–59	1,695 (20.2)		1,270 (20.1)		
60–70†	715 (8.5)		572 (9.1)		
Patient sex					0.01
M	3,535 (42.1)		2,552 (40.4)		
F	4,802 (57.2)		3,700 (58.6)		
Prefer not to answer	55 (0.7)		66 (1.0)		
Mean BMI, kg/m^2^ (+SD)	22.1 (+3.5)		22.3 (+3.8)		0.08
BMI, kg/m^2^					0.001
<18.5	932 (11.1)		757 (12.0)		
18.5–25.0	5,902 (70.3)		4,271 (67.6)		
>25.0	1,406 (16.8)		1,174 (18.6)		
Underlying medical conditions‡					0.01
0	6,445 (76.8)		4,752 (75.2)		
1	1,382 (16.5)		1,057 (16.7)		
>2	565 (6.7)		509 (8.1)		
Hypertension	557 (6.6)		441 (7.0)		0.41
Dyslipidemia	396 (4.7)		362 (5.7)		0.01
Respiratory diseases	394 (4.7)		317 (5.0)		0.37
Depression/anxiety	272 (3.2)		243 (3.8)		0.05
Heart diseases	197 (2.3)		180 (2.8)		0.06
Malignancy	169 (2.0)		131 (2.1)		0.80
Diabetes	152 (1.8)		167 (2.6)		0.001
No. COVID-19 vaccinations§					<0.001
0	685 (8.2)		412 (6.5)		
1	49 (0.6)		28 (0.4)		
2	1,675 (20.0)		1,145 (18.1)		
>3	5,983 (71.3)		4,733 (74.9)		
Household income, ¥					0.002
<4 million	2,520 (34.5)		2,022 (32.0)		
4–8 million	3,699 (50.7)		2,614 (41.4)		
>8 million	1,077 (14.8)		858 (13.6)		
Education level					0.68
High school or lower	1,242 (14.8)		961 (15.2)		
Some college	1,710 (20.4)		1,259 (19.9)		
College or higher	5,299 (63.1)		4,004 (63.4)		
Mean follow-up, d (+SD)	167.9 (+14.5)		NA		
No. SARS-CoV-2 infections					
1	8,284 (98.7)		NA		
2	108 (1.3)		NA		
Severity of infection					
Asymptomatic	228 (2.7)		NA		
Mild	8,098 (96.5)		NA		
Moderate/severe	66 (0.8)		NA		

*Values are no. (%) except as indicated. Continuous variables were compared by using *t*-tests; categorical variables were compared by using χ^2^ tests. BMI, body mass index; NA, not applicable. †Includes patients who turned 70 years of age between the participant selection and survey periods. ‡Respiratory diseases included interstitial lung diseases, asthma, and chronic obstructive pulmonary diseases. Heart diseases included myocardial infarction, angina, heart failure, arrhythmia, myocarditis, and cardiomyopathy. Mental disorder included anxiety and depression. §Number of vaccinations administered until 14 d before infection (cases) or before June 2022 (controls).

**Figure 2:**
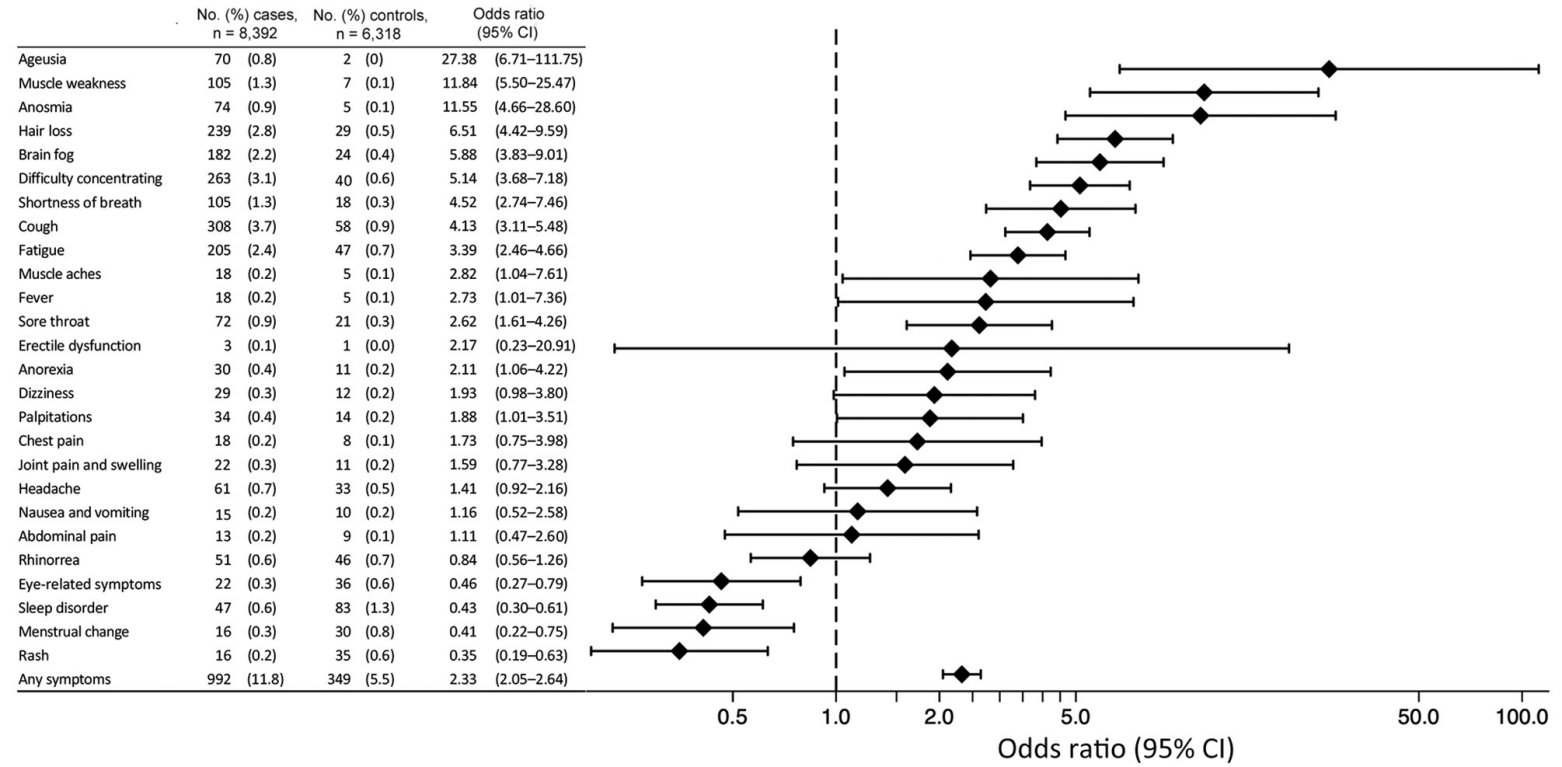
Prevalence and age- and sex-adjusted odds ratios of persistent symptoms in cases compared with controls in study of prevalence and risk factors for post–COVID-19 conditions during Omicron BA.5–dominant wave, Japan. All cases and controls are included in the multivariable logistic regression analysis to estimate the odds ratio of developing post–COVID-19 condition among cases compared with controls adjusting for age (as a continuous variable) and sex.

The percentage of post–COVID-19 condition for cases was 11.8%, whereas the percentage of persistent symptoms among controls was 5.5% (Figure 2). The prevalence did not differ between cases under follow-up for <6 months (11.6%) and cases under follow-up for >6 months (12.6%). The most frequent post–COVID-19 condition was cough (3.7%), followed by difficulty concentrating (3.1%), hair loss (2.8%), fatigue (2.4%), and brain fog (2.2%). The most frequent persistent symptoms among the controls were sleep disorders (1.3%), followed by cough (0.9%), fatigue (0.7%), and rhinorrhea (0.7%). The age- and sex-adjusted odds ratio (OR) of any persistent symptoms for cases versus controls was 2.33 (95% CI 2.05–2.64). Symptoms with higher OR in cases than controls were ageusia (27.4 [95% CI 6.7–111.8]), muscle weakness (11.8 [95% CI 5.5–25.5]), anosmia (11.6 [95% CI 4.7–28.6]), hair loss (6.5 [95% CI 4.4–9.6]), and brain fog (5.9 [95% CI 3.8–9.0]).

We conducted multivariable logistic regression analysis to investigate the factors associated with post–COVID-19 condition among cases (Table 3). In all 3 models, participants 40–49 years of age had higher odds of having post–COVID-19 condition than those 20–29 years (OR 1.26, 95% CI 1.01–1.57 for model 3); female participants had higher odds of having post–COVID-19 condition than male participants (OR 2.00, 95% CI 1.71–2.34). When models were further adjusted, 2 variables were associated with having post–COVID-19 condition: having any underlying medical conditions (OR 1.36, 95% CI 1.16–1.59, compared with no underlying medical conditions), and severity of acute COVID-19 (mild, OR 2.07, 95% CI 1.18–3.66; moderate, OR 4.49, 95% CI 1.97–10.23, compared with asymptomatic). Those participants vaccinated before infection had lower odds of developing post–COVID-19 condition (OR 0.75, 95% CI 0.60–0.95, compared with unvaccinated). Socioeconomic status, including household income and educational level, was not associated with post–COVID-19 condition.

**Table 3:** Factors associated with the prevalence and risk factors for post–COVID-19 conditions during BA.5 Omicron-dominant wave, Japan*

Factor	No. at risk, n = 8,392	No. (%) cases,† n = 992	Model 1			Model 2			Model 3	
			OR (95% CI)	p value		OR (95% CI)	p value		OR (95% CI)	p value
Age group, y										
20–29	1,316	134 (10.2)	Referent	NA		Referent	NA		Referent	NA
30–39	2,340	289 (12.4)	1.31 (1.05–1.63)	0.02		1.23 (0.98–1.54)	0.07		1.22 (0.97–1.52)	0.08
40–49	2,326	307 (13.2)	1.40 (1.12–1.74)	0.003		1.32 (1.06–1.65)	0.01		1.26 (1.01–1.57)	0.05
50–59	1,695	206 (12.2)	1.33 (1.05–1.69)	0.02		1.23 (0.96–1.56)	0.10		1.16 (0.91–1.48)	0.24
60–70	715	56 (7.8)	0.83 (0.60–1.16)	0.28		0.75 (0.53–1.05)	0.10		0.70 (0.50–0.98)	0.04
Patient sex										
M	3,535	280 (7.9)	Referent	NA		Referent	NA		Referent	NA
F	4,802	703 (14.6)	1.98 (1.71–2.30)	<0.001		2.05 (1.76–2.39)	<0.001		2.00 (1.71–2.34)	<0.001
Underlying medical conditions										
Yes	1,947	263 (13.5)	NA	NA		1.36 (1.15–1.60)	<0.001		1.36 (1.16–1.59)	<0.001
No	6,445	729 (11.3)	NA	NA		Referent			Referent	NA
BMI, kg/m^2^										
<18.5	932	119 (12.8)	NA	NA		0.94 (0.76–1.17)	0.59		0.94 (0.76–1.16)	0.58
18.5–25.0	5,902	686 (11.6)	NA	NA		Referent			Referent	NA
>25.0	1,406	162 (11.5)	NA	NA		1.09 (0.90–1.32)	0.36		1.09 (0.90–1.31)	0.39
Severity of acute COVID-19										
Asymptomatic	228	13 (5.7)	NA	NA		Referent			Referent	NA
Mild	8,098	965 (11.9)	NA	NA		2.00 (1.13–3.52)	0.02		2.07 (1.18–3.66)	0.01
Moderate	64	14 (21.9)	NA	NA		4.00 (1.73–9.23)	0.001		4.49 (1.97–10.23)	<0.001
Severe	2	0	NA	NA		NA	NA		NA	NA
Vaccination before infection										
Yes	7,707	890 (11.5)	NA	NA		0.74 (0.59–0.92)	0.01		0.75 (0.60–0.95)	0.02
No	685	102 (14.9)	NA	NA		Referent	NA		Referent	NA
Household income, ¥										
<4 million	2,520	295 (11.7)	NA	NA		NA	NA		Referent	NA
4–8 million	3,699	433 (11.7)	NA	NA		NA	NA		1.05 (0.89–1.25)	0.54
>8 million	1,077	128 (11.9)	NA	NA		NA	NA		1.10 (0.87–1.40)	0.43
Education level										
High school or lower	1,242	137 (11.0)	NA	NA		NA	NA		Referent	NA
Some college	1,710	252 (14.7)	NA	NA		NA	NA		1.20 (0.95–1.50)	0.12
College or higher	5,299	583 (11.0)	NA	NA		NA	NA		1.01 (0.82–1.25)	0.92

*Associations were determined by using multivariable logistic regression models for 8,392 infected persons (cases). Model 1 included age (as a categorical variable) and sex; model 2 added preexisting medical conditions (factor variable), BMI (categorical variable), severity of acute COVID-19 (categorical variable), and vaccination before infection (factor variable); model 3 added household income and education level (categorical variables). BMI, body mass index; NA, not applicable; OR, odds ratio. †Number of cases who had post–COVID-19 condition.

Among the 992 cases who had experienced any post–COVID-19 condition, 84 (8.5%) answered that the condition was a serious hindrance on their daily lives at the time of response. A total of 402 (40.5%) noted that it was no hindrance, 362 (36.5%) mild hindrance, and 144 (14.5%) moderate hindrance.

## Discussion

We conducted a population-based study using a self-reported questionnaire among adults in Japan who had confirmed SARS-CoV-2 infection during July–August 2022, when the Omicron BA.5 subvariant was dominant. We compared their post–COVID-19 condition with concordant persistent symptoms among noninfected controls. The percentage of post–COVID-19 condition was 11.8% for cases, which was 2.3 times higher than the 5.5% of persistent symptoms noted in controls. The cases had a 6.2% higher prevalence of post–COVID-19 condition than the controls, suggesting that their symptoms were likely associated with SARS-CoV-2 infection.

Population-based studies of infected persons in the United Kingdom (n = 56,003) and the United States (n = 1,480) using smartphone applications reported that the prevalence of post–COVID-19 condition associated with the Omicron variant, defined as symptoms lasting 4 weeks after the infection, was 4.5%–18.7% (*12*,*23*). Another population-based study of infected persons in the United States (n = 16,091) showed a prevalence of 11.2% (*16*) applying the WHO definition of the continuation or development of new symptoms 3 months after the initial SARS-CoV-2 infection, with those symptoms lasting for >2 months with no other explanation (*27*). Although the definition of post–COVID-19 condition varies among previous studies (*12**,**16*,*23**,**27*), the proportion shown in our study is consistent with previous results. In those reports, post–COVID-19 condition was less prevalent among those infected during the Omicron variant–dominant wave than those infected during the previous waves with the ancestral strain predominance (*16*,*23*). However, although a multicenter prospective cohort study showed a higher proportion of prolonged severe fatigue and multiple symptoms at 3 months during the pre-Delta wave than that during the Delta and Omicron waves, the differences disappeared after accounting for sociodemographics and vaccination status (*19*). Systematic reviews suggested that vaccination before infection was associated with a lower risk of experiencing post–COVID-19 condition (*30*,*31*). Similarly, we found that vaccination before infection was associated with lesser post–COVID-19 condition. An in-depth study would clarify whether the reduced risk for post–COVID-19 condition during the Omicron wave was a result of the differences in strains, the effect of vaccination, or both. 

Population-based large cohort studies in the United Kingdom (n = 606,434 and n = 486,149) and Germany (n = 11,710) reported that patients infected with previous-variant SARS-CoV-2 frequently experienced persistent symptoms such as fatigue, shortness of breath, concentration difficulties, memory disturbance, hair loss, and anosmia (*5*,*7*,*32*). Studies on patients infected with the Omicron variant, including a population-based study in the United States (n = 16,091) and hospital-based studies from China (n = 1,829) and India (n = 524), revealed that fatigue, brain fog, cough, and shortness of breath were frequently observed as post–COVID-19 condition (*13*,*16*,*33*). Our findings were comparable with previous results; we observed that post–COVID-19 condition after the Omicron-dominant epidemic frequently included neurologic symptoms such as difficulty concentrating, fatigue, and brain fog, in addition to cough and hair loss. In addition, those neurologic symptoms, as well as ageusia, anosmia, and muscle weakness, were distinctive symptoms among cases, who showed a higher OR than controls. Fatigue and neurocognitive impairment are reportedly related to impaired health recovery and reduced working capacity, even among young and middle-aged adults, after mild infection (*7*). Our results showed that ≈10% of those who had post–COVID-19 condition had persistent difficulties in daily living 4.5–7 months after the Omicron-dominant wave, which may have led to a deterioration in economic conditions or work productivity. Although background socioeconomic status was not associated with developing post–COVID-19 condition in this study, further investigation is required to evaluate the effect of post–COVID-19 condition on changes in economic conditions, schooling, and employment.

Large-scale population-based cohort studies on infection before the Omicron wave found that post–COVID-19 condition was more common in female persons, smokers, persons with obesity, those with more severe acute COVID-19 symptoms, and those who were deprived or had lower household income (*5*,*7*,*32*). Moreover, hospital-based studies in China (n = 21,799) and South Africa (n = 4,685) showed that the female sex, concurrent conditions, and severe acute illnesses were associated with post–COVID-19 condition in association with the Omicron variant (*14*,*21*), which was consistent with our findings. Although the results regarding age are unclear, some studies on the Omicron variant have suggested that the population 18–50 years of age has a higher risk for post–COVID-19 condition (*21*,*34*). Our study showed that post–COVID-19 condition for those infected during the Omicron-dominant epidemic was also more prevalent in middle-aged persons. A substantial proportion of the working-age population might have been affected; of 9 million persons infected during July–August 2022 in Japan, 31.2% were in their 30s and 40s (*35*).

The strengths of this study are the large number of participants including noninfected controls, the population-based approach, and the inclusion of all infected residents registered in the HER-SYS database within a municipality. We compared the infected persons with noninfected persons as a control and assessed the proportion of post–COVID-19 condition after the Omicron-dominant wave.

The first limitation of this study is that the response rate was higher among the infected group than the noninfected group overall. The infected participants may have been more interested in the survey on COVID-19 and post–COVID-19 condition. However, because we did not specify the purpose of the survey to investigate the post–COVID-19 condition but rather informed the participants that we aimed to investigate the effect of the pandemic on their health and daily lives, we believe that the influence of interest in post–COVID-19 condition on the responses to the questionnaire was small. Moreover, the response rate was higher for infected and noninfected female participants and middle-aged infected male participants; this finding could have been because those persons were inherently willing to answer questionnaires more than other persons, or because patients with those attributes (such as female sex and middle age) suffered more from persistent symptoms and had a higher motivation to answer the questionnaire. The results could be biased in both ways; however, we believe the effect was small because the higher odds of having post–COVID-19 condition in our study were consistent with findings from previous studies. Second, although we excluded those who self-reported having SARS-CoV-2 infection, it is possible that some infected persons were included in the controls, causing an underestimation of the difference in persistent symptoms between the cases and controls. Third, because the study was retrospective, recall bias may have occurred. In addition, because we relied on self-reporting, we could not rule out the possibility that the participants’ symptoms were caused by conditions other than COVID-19. However, we estimated the symptoms attributable to COVID-19 by comparing with a noninfected control group. Finally, although this study included all infected persons registered in the nationally established registry system, caution is needed to generalize the results of this single-city analysis to other populations in Japan.

In this population-based study, 11.8% of patients with COVID-19 had post–COVID-19 condition during the Omicron-dominant wave; this rate was 2.3 times higher than the persistent symptoms among noninfected controls. Among the cases, female sex, underlying medical conditions, and severity of acute COVID-19 were associated with having post–COVID-19 condition. We recommend a longer follow-up study of the effects on daily life and socioeconomic status after infection during the Omicron-dominant wave.

Appendix Additional information about prevalence and risk factors for post–COVID-19 conditions during Omicron BA.5–dominant wave, Japan.
