# Supplementary material for: Prevalence of and Risk Factors for Post–COVID-19 Condition during Omicron BA.5–Dominant Wave, Japan
Source: Emerg Infect Dis. 2024 Jul;30(7):1380–9. doi: 10.3201/eid3007.231723 (PMC11210664; doi:10.3201/eid3007.231723)
Supplement: Appendix — Additional information about prevalence and risk factors for post–COVID-19 conditions during Omicron BA.5–dominant wave, Japan. [file 23-1723-Techapp-s1.pdf]

Article DOI: http://doi.org/10.3201/eid3007.231723

EID cannot ensure accessibility for supplementary materials supplied by authors. Readers who have difficulty accessing supplementary content should contact the authors for assistance.

Prevalence and Risk Factors of Post–COVID-19 Condition during Omicron BA.5–Dominant Wave, Japan

Appendix

Shown on the following pages is the questionnaire sent to residents in Shinagawa City, a metropolitan area located in the Tokyo area of Japan, who were 20–69 years of age and infected with SARS-CoV-2 during July 1–August 31, 2022, along with noninfected residents as controls (25,911 in each group).

Appendix

Questionnaire (written in green indicates directions)

Survey on the health of residents during COVID-19 pandemic		
	The purpose of this survey is to ask about your health and daily life during the Coronavirus Disease 2019 (COVID-19) pandemic and the recovery status of those who have had COVID-19 in order to provide reference for future healthcare systems.<br>Please read the enclosed "Regarding Cooperation with the 'Survey on the health of residents during COVID-19 pandemic,'" and if you agree to cooperate with this survey, please click the "I agree" button to proceed to the answer section.<br>This work was supported by MHLW Research on Emerging and Re-emerging Infectious Diseases and Immunization (Program Grant Number JPMH21HA2011).	I agree.
Section 1 (questionnaire for all participants)		
Q	Respondent	1=the person himself/herself<br>2=substitute
Q	Sex (of the person himself/herself)	1=male<br>2=female<br>3=I prefer not to answer
Q	Age (of the person himself/herself)	year
Q	Have you ever been diagnosed to be a close contact with someone who had COVID-19?	1=Yes<br>2=No<br>3=I don't know<br>4=I prefer not to answer
Q	Has anyone living with you been diagnosed with COVID-19	1=Yes<br>2=No

		3=No roommate (living alone)<br>4=I don't know<br>5=I prefer not to answer
Q	Have you ever been diagnosed to have COVID-19?<br>Yes → move to the questionnaire for infected persons; other answers → move to the questionnaire for noninfected persons	1=Yes<br>2=No<br>3=I don't know<br>4=I prefer not to answer

	Section 2-A (questionnaire for infected persons)	
Q	How many times have you been diagnosed with COVID-19?	Once<br>Twice<br>Three times or more
	(Show only the number of times diagnosed) When was the date of diagnosis?<br>If you do not know the exact date, please provide an approximate date.	Choose from below<br>B: 2019, 2020, 2021, 2022, 2023<br>C: January–December<br>D: 1–31, unknown
Q	How was your medical condition when you had COVID-19 for the first time?	
	Did you have any symptoms?	1=Yes 2=No 3=I don't know
	Were you diagnosed with pneumonia?	1=Yes 2=No 3=I don't know
	Were you hospitalized?	1=Yes 2=No 3=I don't know

First diagnosis date		B year	C month	D day
Second diagnosis date		B year	C month	D day
Third diagnosis date		B year	C month	D day

	<Those reported being hospitalized>Were you admitted to the intensive care unit (ICU)?	1=Yes 2=No 3=I don’t know
	Was oxygen administered?	1=Yes 2=No 3=I don’t know
	<Those with oxygen administration>Please select all methods of oxygen administration received at that time.<br>1 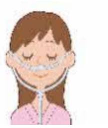 2 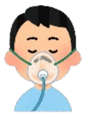 3 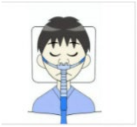 4 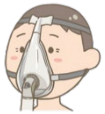 5 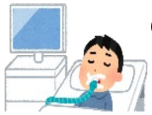 6 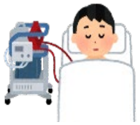	1=Nasal cannula<br>2=oxygen mask<br>3=high-flow nasal cannula<br>4=noninvasive positive pressure ventilation (NPPV)<br>5=mechanical ventilation<br>6=extracorporeal membrane oxygenation (ECMO)<br>7=Oxygen was administered, but I don’t know how.
Q	How was your medical condition when you had COVID-19 for the second time?	
	Did you have any symptoms?	1=Yes 2=No 3=I don’t know
	Were you hospitalized?	1=Yes 2=No 3=I don’t know
	Was oxygen administered?	1=Yes 2=No 3=I don’t know
	Were you intubated (mechanical ventilation)?	1=Yes 2=No 3=I don’t know
Q	How was your medical condition when you had COVID-19 for the third time?	
	Did you have any symptoms?	1=Yes 2=No 3=I don’t know
	Were you hospitalized?	1=Yes 2=No 3=I don’t know
	Was oxygen administered?	1=Yes 2=No 3=I don’t know
	Were you intubated (mechanical ventilation)?	1=Yes 2=No 3=I don’t know
Q	<Female only>Were you pregnant when you had COVID-19? (multiple choices)	1=Yes 2=No 3=I don’t know 4=I prefer not to answer

	<Those who answered yes, multiple choice>During which infection episode were you pregnant?	First infection, second infection, third infection, other
	<Those who answered yes>What was the gestational age at the time of the first infection?	1=First trimester (≦15 weeks gestation)<br>2=Second trimester (≧16weeks and ≦27 weeks)<br>3=Third trimester (≧28 weeks)<br>4=I don't know the gestational age at the time of infection
	<Those who answered yes>What was the gestational age at the time of the second infection?	1=First trimester (≦15 weeks gestation)<br>2=Second trimester (≧16weeks and ≦27 weeks)<br>3=Third trimester (≧28 weeks)<br>4=I don't know the gestational age at the time of infection
	<Those who answered yes>What was the gestational age at the time of the third infection?	1=First trimester (≦15 weeks gestation)<br>2=Second trimester (≧16weeks and ≦27 weeks)<br>3=Third trimester (≧28 weeks)<br>4=I don't know the gestational age at the time of infection
Q	We would like to ask you about the symptoms that appeared after the COVID-19 diagnosis.<br>Please select the symptoms that existed between the COVID-19 diagnosis in the summer of 2022 to date.<br>Please check all that apply regarding the duration of the symptoms.	<Selecting symptoms will display "Was present from the time of infection through the recovery period", "Was present at 3 months after the

- If the symptoms were present from the time of infection through the recovery period, check “Was present from the time of infection through the recovery period”
- If the symptoms were present at 3 months after the infection, check “Was present after three months of infection”
- If the symptom lasted 2 months or more, check “lasted 2 months or more”
- If the symptom is present, check “Still present”

If none of the symptoms were present, please select “No symptoms of any of the above” at the bottom.

*For those who had COVID-19 more than once, please answer the following questions regarding the infection in the summer of 2022.

Symptoms that have existed between the COVID-19 diagnosis in the summer of 2022 to date	Symptom	Was present from the time of infection through the recovery period	Was present at 3 months after the infection	Lasted 2 months or more	Still present
□	Fever (≥37.5 °C)	□	□	□	□

infection”, “Lasted 2 months or more”, and “Still present”.>

	☐	Cough	☐	☐	☐	☐		
	☐	Fatigue	☐	☐	☐	☐		
	☐	Sore throat	☐	☐	☐	☐		
	☐	Chest pain	☐	☐	☐	☐		
	☐	Lack of appetite (anorexia)	☐	☐	☐	☐		
	☐	Brain fog<br>(Brain fog is a state such as being “foggy,” “confused,” “having difficulty learning new things,” “indecisive,” or “unable to think clearly.”)	☐	☐	☐	☐		
	☐	Difficulty in concentrating	☐	☐	☐	☐		
	☐	Loss of smells (smelling disorder)	☐	☐	☐	☐		
	☐	Loss of tastes (tasting disorder)	☐	☐	☐	☐		
	☐	Shortness of breath	☐	☐	☐	☐		
	☐	Hair loss	☐	☐	☐	☐		
	☐	Muscle weakness	☐	☐	☐	☐		
	☐	Palpitations	☐	☐	☐	☐		

	☐	Difficulty sleeping, waking up at night or early in the morning (sleep disorder)	☐	☐	☐	☐	
	☐	Runny nose	☐	☐	☐	☐	
	☐	Headache	☐	☐	☐	☐	
	☐	Joint pain or swelling	☐	☐	☐	☐	
	☐	Muscle pain	☐	☐	☐	☐	
	☐	Nausea and vomiting	☐	☐	☐	☐	
	☐	Abdominal pain	☐	☐	☐	☐	
	☐	Skin rash	☐	☐	☐	☐	
	☐	Eye symptoms (eye pain, itching, foreign body sensation, redness, watery eyes, blurred vision, difficulty seeing)	☐	☐	☐	☐	
	☐	Dizziness	☐	☐	☐	☐	
	☐	Erectile disfunction (male only)	☐	☐	☐	☐	
	☐	Menstrual change (female only)	☐	☐	☐	☐	
	☐	Other 1 （in detail)	☐	☐	☐	☐	

☐	Other 2 (in detail<br>)	☐	☐	☐	☐
☐	No symptoms of any of the above				

Q	To what extent are the symptoms after COVID-19 you selected in the previous question currently interfering with your daily life?<br>Please choose a number from 0 to 10, with 0 being "no hindrance at all" and 10 being "extreme hindrance."	0 to 10
Q	Are you currently receiving treatment for any of the symptoms selected in the previous question?	1=Yes<br>2=No
	<Those who answered yes> Please select all treatments you are currently receiving.	1=in-patient care<br>2=out-patient care<br>3=prescription drugs (other than herbal medicines)<br>4=prescription drugs (herbal medicines)<br>5=over-the-counter drug<br>6=rehabilitation<br>7=psychological counseling<br>8=other ( )
Q	Please select all new diseases diagnosed at medical facilities after COVID-19 diagnosis in the summer of 2022.	1=Malignancy<br>2=Myocardial infarction, angina<br>3=Heart failure<br>4=Arrhythmia<br>5=Myocarditis/cardiomyopathy

		6=Stroke<br>7=Hypertension<br>8=Hyperlipidemia<br>9=Gout (Hyperuricemia)<br>10–12=Diabetes (Type 1, type 2, I don't know if it's type 1 or 2)<br>13=Interstitial pneumonia/interstitial lung disease<br>14=Asthma<br>15=Pulmonary embolism<br>16=Chronic obstructive pulmonary disease (COPD)<br>17=Hepatitis/cirrhosis<br>18–19=Chronic kidney disease (with dialysis, without dialysis)<br>20=Connective tissue disease<br>21=Shingles<br>22=Dementia<br>23=Depression<br>24=Anxiety<br>25=Other<br>0=None

	Section 2-B (questionnaire for noninfected persons)	

Q	We would like to ask you about the symptoms that appeared after July 2022.<br>Please select the symptoms that existed between July 2022 to date.<br>• If the symptom lasted 2 months or more, check "lasted 2 months or more"<br>• If the symptom is present, check "Still present"<br>If none of the symptoms were present, please select "No symptoms of any of the above" at the bottom.	

Symptoms that have existed from July 2022 to date	Symptom	Lasted 2 months or more	Still present
☐	Fever (≥37.5 °C)	☐	☐
☐	Cough	☐	☐
☐	Fatigue	☐	☐
☐	Sore throat	☐	☐
☐	Chest pain	☐	☐
☐	Lack of appetite (anorexia)	☐	☐
☐	Brain fog<br>(Brain fog is a state such as being "foggy," "confused," "having difficulty learning new things," "indecisive," or "unable to think clearly.")	☐	☐
☐	Difficulty in concentrating	☐	☐

	☐	Loss of smells (smelling disorder)	☐	☐	
	☐	Loss of tastes (tasting disorder)	☐	☐	
	☐	Shortness of breath	☐	☐	
	☐	Hair loss	☐	☐	
	☐	Muscle weakness	☐	☐	
	☐	Palpitations	☐	☐	
	☐	Difficulty sleeping, waking up at night or early in the morning (sleep disorder)	☐	☐	
	☐	Runny nose	☐	☐	
	☐	Headache	☐	☐	
	☐	Joint pain or swelling	☐	☐	
	☐	Muscle pain	☐	☐	
	☐	Nausea and vomiting	☐	☐	
	☐	Abdominal pain	☐	☐	
	☐	Skin rash	☐	☐	
	☐	Eye symptoms (eye pain, itching, foreign body sensation, redness, watery eyes, blurred vision, difficulty seeing)	☐	☐	
	☐	Dizziness	☐	☐	
	☐	Erectile disfunction (male only)	☐	☐	
	☐	Menstrual change (female only)	☐	☐	

	☐	Other 1 (in detail)	☐	☐	
	☐	Other 2 (in detail)	☐	☐	
	☐	No symptoms of any of the above	☐	☐	
Q	Please select all new diseases diagnosed at medical facilities after July 2022.				1=Malignancy<br>2=Myocardial infarction, angina<br>3=Heart failure<br>4=Arrhythmia<br>5=Myocarditis/cardiomyopathy<br>6=Stroke<br>7=Hypertension<br>8=Hyperlipidemia<br>9=Gout (Hyperuricemia)<br>10–12=Diabetes (Type 1, type 2, I don't know if it's type 1 or 2)<br>13=Interstitial pneumonia/interstitial lung disease<br>14=Asthma<br>15=Pulmonary embolism<br>16=Chronic obstructive pulmonary disease (COPD)<br>17=Hepatitis/cirrhosis<br>18–19=Chronic kidney disease (with dialysis, without dialysis)<br>20=Connective tissue disease

		21=Shingles<br>22=Dementia<br>23=Depression<br>24=Anxiety<br>25=Other<br>0=None
	<Female only>Have you been pregnant between July 2022 to date?	1=Yes 2=No 3=I don't know<br>4=I prefer not to answer

Section 3 (questionnaire for all participants)

	We would like to ask about COVID-19 vaccines.	
Q	How many doses of COVID-19 vaccine have you received so far?	0 (unvaccinated), once, twice, three times, four times or more
Q	Please indicate the date of vaccination.<br>If you do not know the exact date, please provide an approximate date.<br>(Displayed according to the number of vaccinations selected)<br>First dose : A year B month<br>Second dose : A year B month<br>Third dose : A year B month<br>Fourth dose : A year B month	Choose from below<br>A: 2020, 2021, 2022, 2023<br>B : January–December

	We would like to ask about your health and daily life.	
Q	Current height	cm

Q	Current weight	kg
Q	Weight about 6 months ago<br>For infected persons: Weight about 6 months ago (before COVID-19 diagnosis in summer 2022)	kg
Q	Do you currently have "breathlessness"? Please choose one that comes closest.	1=No breathlessness<br>2=I only get breathless with strenuous exercise<br>3=I get short of breath when hurrying on level ground or walking up a slight hill<br>4=On level ground, I walk slow than people of my age because of breathlessness, or I have to stop for breath when walking at my own pace on the level<br>5=I stop for breath after walking about 100 m or after a few minutes on level ground<br>6=I am too breathless to leave the house or I am breathless when dressing/undressing
Q	Do you currently have "fatigue"? Please choose one that comes closest. If you do not have "fatigue," please choose "Fully active."	1=Fully active, able to carry on all (infected persons: pre-disease) performance without restriction<br>2=Restricted in physically strenuous activity but ambulatory and able to carry out work of a light or sedentary nature, e.g., light house work, office work<br>3=Ambulatory and capable of all selfcare but unable to carry out any work activities. Up and about more than 50% of waking hours<br>4=Capable of only limited selfcare, confined to bed or chair more than 50% of waking hours

		**5**=Completely disabled. Cannot carry on any selfcare. Totally confined to bed or chair
Q	Please select all diseases diagnosed at medical institutions before July 2022.<br>For infected persons: Please select all diseases diagnosed at medical facilities prior to the COVID-19 infection in the summer of 2022.	1=Malignancy<br>2=Myocardial infarction, angina<br>3=Heart failure<br>4=Arrhythmia<br>5=Myocarditis/cardiomyopathy<br>6=Stroke<br>7=Hypertension<br>8=Hyperlipidemia<br>9=Gout (Hyperuricemia)<br>10–12=Diabetes (Type 1, type 2, I don't know if it's type 1 or 2)<br>13=Interstitial pneumonia/interstitial lung disease<br>14=Asthma<br>15=Pulmonary embolism<br>16=Chronic obstructive pulmonary disease (COPD)<br>17=Hepatitis/cirrhosis<br>18–19=Chronic kidney disease (with dialysis, without dialysis)<br>20=Connective tissue disease<br>21=Shingles<br>22=Dementia<br>23=Depression<br>24=Anxiety

		25=Other<br>0=None
Q	On or about July 2022 (infected persons: at the time of COVID-19 diagnosis in summer 2022), were you treated with steroids (excluding ointments), anticancer drugs, immunosuppressive drugs or biologics (such as Remicade)?	1=Yes<br>2=No<br>3=I don't know
Q	Do you smoke cigarettes (cigarettes, heated tobacco products, e-cigarettes including nicotine)?	1=Never smoked cigarettes habitually before<br>2=Smoked cigarettes in the past but have quit<br>3=Currently still smoking cigarettes
	<Those who answered to have quit><br>When did you quit smoking cigarettes?	1=Before COVID-19 diagnosis (noninfected persons: before July 2022)<br>2=After COVID-19 diagnosis (noninfected persons: after July 2022)
	<Those who answered currently smoking><br>Has the amount of cigarettes you smoke changed compared with that 6 months ago (infected persons: 6 months ago (before COVID-19 infection in summer 2022))?	1=Decreased<br>2=Unchanged<br>3=Increased
Q	How often do you currently drink alcohol (alcohol-containing beverages)?	1=I don't drink at all (can't drink)<br>2=Hardly drink (about once a month)<br>3=1–3 times a month<br>4=1–2 times a week<br>5=3–4 times a week<br>6=5–6 times a week<br>7=every day

	<Those who chose other than "don't drink at all" above><br>What is the average amount of alcohol you drink per day? Please choose one that applies to you in terms of sake.	1=Less than 1 cup (180ml) of sake<br>2=1 cup of sake<br>3=2 cups of sake<br>4=3–4 cups of sake<br>5=5 cups of sake
	Compared with that 6 months ago (infected person: 6 months ago (before COVID-19 infection in summer 2022)), has the amount of alcohol you drink changed?	1=I quit<br>2=Decreased<br>3=Unchanged<br>4=Increased (started drinking)
Q	Please select your current average daily sleep duration.	1=less than 5 hours<br>2=5 hours<br>3=6 hours<br>4=7 hours<br>5=8 hours<br>6=9 hours<br>7=10 hours or more
Q	Compared with that 6 months ago (infected persons: 6 months ago (before COVID-19 infection in summer 2022)), has your sleeping duration changed?	1=Decreased<br>2=Unchanged<br>3=Increased
Q	How much sports or exercise do you currently do on average per week in total?	5=7 hours or more<br>4=5–6 hours<br>3=3–4 hours<br>2=1–2 hours<br>1=seldom or never

<table>
<tr><td>Q</td><td>How much time do you currently spend walking indoors or outdoors on average per day?</td><td>4=3 hours or more
3=about 2 hours
2=about 1 hour
1=about 30 minutes
0=seldom or never</td></tr>
<tr><td>Q</td><td>Compared with that 6 months ago (infected persons: 6 months ago (before COVID-19 infection in summer 2022)), have you changed the way you move your body or how long you sit?
<table>
<tr><th></th><th>Decreased</th><th>Unchanged</th><th>Increased</th></tr>
<tr><td>Frequency and duration of sports and exercise</td><td>□</td><td>□</td><td>□</td></tr>
<tr><td>Time spent walking indoors and outdoors</td><td>□</td><td>□</td><td>□</td></tr>
<tr><td>Sitting time</td><td>□</td><td>□</td><td>□</td></tr>
</table>
</td><td></td></tr>
<tr><td>Q</td><td><For those aged 50 years or older only>
Please select “Yes” or “No” for the following five items.</td><td></td></tr>
</table>

	Yes	No
Have you lost 2 kg or more in the past 6 months?	□	□
Do you think you walk slower than before?	□	□
Do you go for a walk for your health at least once a week?	□	□
Can you recall what happened 5 minutes ago?	□	□
In the past 2 weeks, have you felt tired without a reason?	□	□

Q For each item, please indicate how often you feel.

	None of the time	Seldom of the time	Often	All of the time
How often do you feel that you lack companionship?	□	□	□	□
How often do you feel left out?	□	□	□	□
How often do you feel isolated from others?	□	□	□	□

<table>
<tr><td>Q</td><td>During the past 30 days, about how often did you feel …

	None of the time	A little of the time	Some of the time	Most of the time	All of the time
…nervous?	□	□	□	□	□
…hopeless?	□	□	□	□	□
…restless or fidgety?	□	□	□	□	□
…so depressed that nothing could cheer you up?	□	□	□	□	□
…that everything was an effort?	□	□	□	□	□
…worthless?	□	□	□	□	□
</td><td></td></tr>
<tr><td>Q</td><td>Over the last 2 weeks, how often have you been bothered by thoughts that you would be better off dead or of hurting yourself in some way?</td><td>1=Not at all
2=Several days
3=More than half the days
4=Nearly every day</td></tr>
</table>

Q	What is your current marital status?	1=Unmarried<br>2=Married<br>3=Widowed<br>4=Divorced<br>5=Prefer not to answer
Q	Who do you currently live with? Please check all persons living with you.	1=Spouse<br>2=Child<br>3=Parent<br>4=Other ( )
Q	<Those who check the above question><br>How many people are currently living with you (not including you)?	persons
	Of the number of people living with you above, how many are under the age of 18 (not including you)?	persons
Q	What was the last school you graduated from?	1=Junior high school<br>2=High school<br>3=Vocational school<br>4=Some college<br>5=University<br>6=Graduate school<br>7=Prefer not to answer
Q	What is your household income (including taxes and income from pensions and dividends) in 2021?	1=No household income<br>2=< 500,000 yen<br>3=≥500,000 yen and < 1,000,000 yen<br>4=≥1,000,000 yen and < 2,000,000 yen<br>5=≥2,000,000 yen and < 4,000,000 yen

		6＝≥4,000,000 yen and <6,000,000 yen<br>7＝≥6,000,000 yen and <8,000,000 yen<br>8＝≥8,000,000 yen and <10,000,000 yen<br>9＝≥10,000,000 yen and <12,000,000 yen<br>10＝≥12,000,000 yen and <14,000,000 yen<br>11＝≥14,000,000 yen<br>12＝Prefer not to answer/don't know
Q	Has your financial situation changed compared with that 6 months ago (infected persons: 6 months ago (before COVID-19 infection in summer 2022))?	1＝Very worse<br>2＝Worse<br>3＝Unchanged<br>4＝Better<br>5＝Very better
Q	Please select one of your main occupations at present. If you have more than one occupation, please select one main job.	1= Administrative and managerial<br>2=Professional and engineering (healthcare)<br>3=Professional and engineering （nursing care/welfare）<br>4=Professional and engineering （childcare/education ）<br>5= Professional and engineering （other）<br>6=Clerical<br>7=Service (accommodation industry)<br>8=Service (restaurant business serving alcohol beverage)<br>9=Service (restaurant business without serving alcohol beverage)

		10=Service (entertainment)<br>11=Service (other)<br>12=Sales (retail store owners, sales clerks, etc.)<br>13=Security<br>14=Manufacturing process<br>15=Agricultural, forestry and fishery<br>16=Transport and machine operation<br>17=Construction and mining<br>18=Carrying, cleaning, packaging, and related<br>19=Student<br>20=Housewife/househusband<br>21=Not apply to the above<br>22=Unemployed
Q	Please select one of your current employment statuses (including student). If you have more than one type of employment, please choose one main status.	1=Regular employee (manager)<br>2=Regular employee (other than managers)<br>3=Executive of company or corporation<br>4=Self-employed worker<br>5=Assistance in private business<br>6=Dispatched worker from temporary labor agency<br>7=Contract employee<br>8=Part-time worker<br>9=Doing piecework at home<br>10=Students<br>11=Housewife/househusband

		12=Unemployed<br>13=Other ( )
Q	<Display selected occupations> Is the occupation here the same as the occupation as of July 1, 2022?	1=Yes<br>2=No
	<Those who answered no><br>Please select your occupation as of July 1, 2022. If you have more than one occupation, please select one main occupation.	1= Administrative and managerial<br>2=Professional and engineering (healthcare)<br>3=Professional and engineering (nursing care/welfare)<br>4=Professional and engineering (childcare/education)<br>5= Professional and engineering (other)<br>6=Clerical<br>7=Service (accommodation industry)<br>8=Service (restaurant business serving alcohol beverage)<br>9=Service (restaurant business without serving alcohol beverage)<br>10=Service (entertainment)<br>11=Service (other)<br>12=Sales (retail store owners, sales clerks, etc.)<br>13=Security<br>14=Manufacturing process<br>15=Agricultural, forestry and fishery<br>16=Transport and machine operation<br>17=Construction and mining

		18=Carrying, cleaning, packaging, and related<br>19=Student<br>20=Housewife/ househusband<br>21=Not apply to the above<br>22=Unemployed
Q	<<Display selected employment status><br>Is the employment status here the same as of July 1, 2022?	1＝Yes<br>2＝No
	<Those who answered no><br>Please select your employment status as of July 1, 2022. If you have more than one employment status, please select one main status.	1=Regular employee (manager)<br>2=Regular employee (other than managers)<br>3=Executive of company or corporation<br>4=Self-employed worker<br>5=Assistance in private business<br>6=Dispatched worker from temporary labor agency<br>7=Contract employee<br>8=Part-time worker<br>9=Doing piecework at home<br>10=Students<br>11=Housewife/ househusband<br>12=Unemployed<br>13=Other ( )
Q	In the past 6 months, have you experienced any of the following?<br>Please select all that apply.	1＝Working (schooling) hours increased<br>2＝Working (schooling) hours decreased<br>3＝ Telecommuting (online classes) has increased<br>4＝Telecommuting (online classes) has decreased<br>5＝I have found a job (entered to school)

		6＝I took a leave of absence<br>7＝Changed jobs (schools)<br>8＝Retired (withdrew from school)<br>9＝I've been frequently absent<br>10＝Other（　　）<br>11＝None of the above
	<Those who selected No.1–10 above><br>Please select all applicable reasons.	1=Because I was instructed by my employer or school<br>2=Due to deteriorating health conditions caused by COVID-19<br>3=Due to deteriorating health conditions caused by conditions other than COVID-19<br>4=Due to a new need for childcare or nursing care for a family member, etc.<br>5=For financial reasons<br>6=Because I could no longer pay my tuition fees<br>7=Other（　　　　　　　　　　）
